# Supplementary material for: Targeting Amino Acid Metabolic Reprogramming via L-Type Amino Acid Transporter 1 (LAT1) for Endocrine-Resistant Breast Cancer
Source: Cancers (Basel). 2021 Aug 30;13(17):4375. doi: 10.3390/cancers13174375 (PMC8431153; doi:10.3390/cancers13174375)
Supplement: Supplementary file 1 [file cancers-13-04375-s001.zip › cancers-1327385.pdf]

Fig. 4(a) (LAT1)

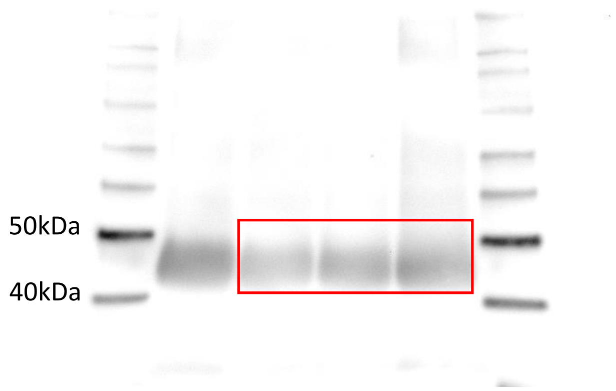

Fig. 4(a) (β-actin)*

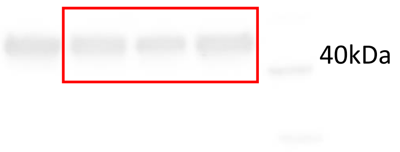

Fig. 4(b) (LAT3)

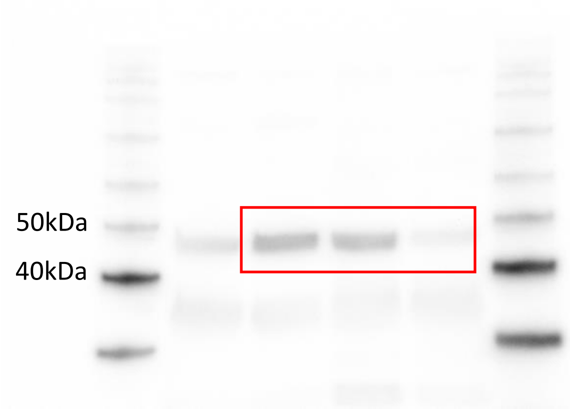

Fig. 4(b) (β-actin)**

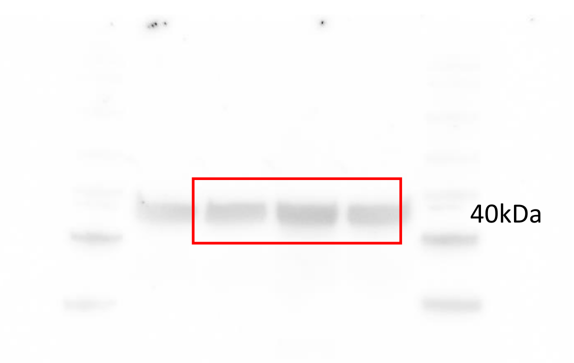

* β-actin is the same sample of LAT1.

** β-actin is the same membrane of LAT3 that was washed and reprobed.
